# Recent Developments of TiO_2_-Based Photocatalysis in the Hydrogen Evolution and Photodegradation: A Review

**DOI:** 10.3390/nano10091790

**Published:** 2020-09-09

**Authors:** Baglan Bakbolat, Chingis Daulbayev, Fail Sultanov, Renat Beissenov, Arman Umirzakov, Almaz Mereke, Askhat Bekbaev, Igor Chuprakov

**Affiliations:** 1 Faculty of Chemistry and Chemical Technology, al-Farabi Kazakh National University, Almaty 050000, Kazakhstan; baglan.bakbolat@mail.ru; 2 Laboratory of Energy-Intensive and Nanomaterials, Institute of Combustion Problems, Almaty 050000, Kazakhstan; 3 Department of Engineering Physics, Satbayev University, Almaty 050013, Kazakhstan; 4 Laboratory of EPR Spectroscopy Named after Y.V. Gorelkinsky, LLP “Institute of Physics and Technology”, Almaty 050032, Kazakhstan; arman_umirzakov@mail.ru (A.U.); mereke.almaz@mail.ru (A.M.); 5 Faculty of Physics and Technology, al-Farabi Kazakh National University, Almaty 050000, Kazakhstan; 6 Frank Laboratory of Neutron Physics, Joint Institute for Nuclear Research (JINR), Dubna 141980, Russia; chupa@nf.jinr.ru; 7 Faculty of Physics and Technical Sciences, L.N. Gumilyov Eurasian National University, Astana 010000, Kazakhstan

**Keywords:** titanium dioxide, photocatalysis, hydrogen evolution, photodegradation

## Abstract

The growth of industrialization, which is forced to use non-renewable energy sources, leads to an increase in environmental pollution. Therefore, it is necessary not only to reduce the use of fossil fuels to meet energy needs but also to replace it with cleaner fuels. Production of hydrogen by splitting water is considered one of the most promising ways to use solar energy. TiO_2_ is an amphoteric oxide that occurs naturally in several modifications. This review summarizes recent advances of doped TiO_2_-based photocatalysts used in hydrogen production and the degradation of organic pollutants in water. An intense scientific and practical interest in these processes is aroused by the fact that they aim to solve global problems of energy conservation and ecology.

## 1. Introduction

At present, hydrogen is regarded as the fuel of the future. Compared to carbon fuel, hydrogen is considered a renewable and environmentally friendly source of energy. There are various methods for producing hydrogen on an industrial scale. However, all known methods are characterized by high energy consumption, which makes the process of hydrogen production on a large scale disadvantageous. The production of hydrogen by photocatalytic water splitting is technologically simple, and the outcoming gases are environmentally friendly.

**Figure 1:**
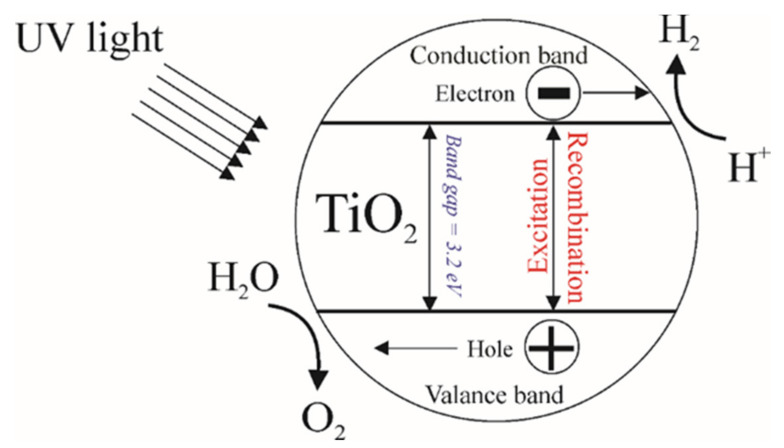
The mechanism of photocatalytic water splitting on TiO_2_ based photocatalyst.

TiO_2_ is a wide-bandgap semiconductor. In nature, TiO_2_ is usually found in three different crystalline structures: rutile, anatase, and brookite. TiO_2_ in anatase form is the most widespread photocatalyst for hydrogen evolution [1]. However, it cannot be used in the spectrum of visible light, since its bandgap (Eg) for different crystalline phases (anatase—3.2 eV, rutile—3.0 eV, and brookite—3.3 eV) is in the UV region. Also, the efficiency of photocatalysis in addition to the bandgap depends on many other factors [2]. In the 1970s, Fujishima and Honda studied the photoelectrochemical splitting of water, where a TiO_2_-based electrode demonstrated the ability to split water under the influence of ultraviolet radiation [3]. Photocatalysis is a complex reaction consisting of the processes starting from light absorption to generate charge carriers up to surface catalytic reactions due to which gas is formed (Figure 1). This process only requires a photocatalyst (TiO_2_), which is not consumed during the entire process, water, sunlight or ultraviolet radiation. During the absorption of light (≥$E_{g}$) by the photocatalyst, an excited electron ($e_{CB}^{-}$) transfers from the valence band into the conduction band. This transition of the electron leads to the generation of a positively charged carrier-hole ($h_{VB}^{+}$) in the valence band (Equation (1)). Also, these charge carriers can recombine among themselves (Equation (2)) [4,5].

(1) $$TiO_{2}\;\overset{hv\geq E_{g}}{\to }\;h_{VB}^{+}+\;e_{CB}^{-}$$

(2) $$h_{VB}^{+}+\;e_{CB}^{-}\;\overset{recombination}{\to }\;Energy$$

Another important application of TiO_2_-based photocatalysts is based on their ability to discolor and completely decompose organic dyes contained in water. In addition, there are a number of works in which TiO_2_-based photocatalysts were used to neutralize harmful to the atmosphere gases [6,7,8,9]. Chemical stability, easy accessibility, nontoxicity and the ability to oxidize under the influence of radiation, allow TiO_2_-based photocatalysts to solve the main global problems associated with pollution of the environment and need for renewable energy [10,11]. Currently, TiO_2_ as a photocatalyst is commercially produced in powder form. The most common brand of TiO_2_ based photocatalyst are P-25 Degussa/Evonik, TiO_2_ nanofibers from Kertak, TiO_2_ Millennium PC-500, TiO_2_ Hombikat UV-100, Sigma Aldrich TiO_2_, TiO_2_ PK-10, and P90 Aeroxide.

This paper aims to provide a brief overview of articles published starting from 2017 on new research and developments of TiO_2_ based photocatalysts with significant advances. In this review, attention is also paid to the study on the mechanism of photocatalytic processes, factors affecting the activity of photocatalysts, and new techniques used to increase the activity of TiO_2_ based photocatalysts during the splitting of water with the evolution of hydrogen and decomposition of organic compounds utilized for water purification.

## 2. Factors Affecting the Efficiency of Photocatalysis and Techniques Used to Improve the Efficiency of TiO_2_-Based Photocatalysts

### 2.1. Lifetime of Photogenerated Charge Carriers

The activity of photocatalysts strongly depends on the lifetime of photogenerated electron-hole pairs. An important role is played by the rate at which charge carriers can reach the surface of the photocatalysts. The results of spectroscopic studies show that the time intervals between redox reactions or recombination involving charge carriers are extremely short, resulting in a significant reduction of the photocatalytic activity of TiO_2_. In the case of recombination of charge carriers in a sufficiently fast interval (<0.1 ns), the photocatalytic activity of the semiconductor is not observed. For example, the lifetime of electron-hole pairs of ~250 ns (TiO_2_) is considered relatively long [12]. Thus, it can be concluded that a high recombination rate and barriers, that prevent the transfer of charge carriers to the semiconductor surface, reduce photoactivity, despite the high concentration of initially photogenerated pairs. In this regard, to avoid their recombination, it becomes necessary to use cocatalysts in order to increase the lifetimes of electrons and holes.

### 2.2. The Particle Size of Photocatalyst

**Figure 2:**
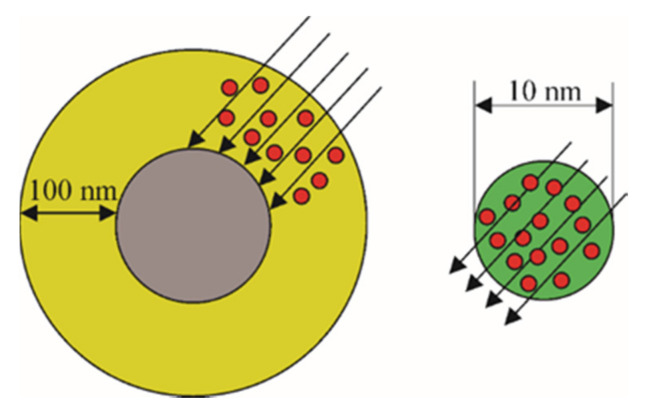
Light absorption by micro- and nanoparticles of TiO_2_.

Compared to microparticles, TiO_2_ nanoparticles have, generally, a higher photocatalytic activity [13,14]. This is due to the small diameter of the nanoparticles, in which the charge requires minimal effort to transfer to the surface. If the particle size decreases, the distance that photogenerated electrons and holes need to travel to the surface where the reactions take place is reduced, thereby reducing the probability of recombination. For TiO_2_ photocatalyst microparticles, the penetration depth of UV rays is limited and amounts to about ~100 nm. This means that the inner part of the TiO_2_ photocatalyst microparticle remains in a passive state. [15]. Figure 2 shows the scheme of light absorption by nano- and microparticles of TiO_2_. This is one of the reasons for the increased interest in nanosized particles of TiO_2_.

As shown in Figure 2, a decrease in the particle size of the photocatalyst to nanoscale facilitates the absorption of light by the entire volume of particles. However, there is a limitation regarding the minimum sizes to which it is desirable to reduce the particles of the photocatalyst, due to the onset of quantum effects. They become significant at particle sizes less than 2 nm for both anatase and rutile, and finally, this leads to a change in the bandgap. An increase in the size of the photocatalyst crystals leads to a decrease in the recombination of the electron-hole pair at the defects of the crystal lattice and to an increase in photocatalytic activity. For example, in [16], nanoparticles with a size of 25 nm were found to be more productive than nanoparticles of 15 nm. On the other hand, the bandgap is directly proportional to the size of the photocatalyst crystals. That is why it is necessary to find the optimal crystal size of TiO_2_ and control it in the process of its obtaining.

To increase the photoactivity of TiO_2_ in the visible region of the spectrum, the spectral region of its absorption should be expanded. There are several approaches for sensitizing TiO_2_ to visible light: doping with cationic and anionic elements or metal nanoparticles. Elements of the 3d- and 2p-groups are often used as additives to reduce the value of the bandgap of the photocatalyst [17,18].

### 2.3. Doping with Cations

The essence of cationic doping is the introduction of metal cations into the crystal structure of TiO_2_ at the position of Ti^4+^ ions. Rare-earth, noble, and transition metal cations can be used [19]. Doping with cations significantly expands the absorption spectrum of TiO_2_, increases the redox potential of the formed radicals, and increases quantum efficiency by reducing the degree of recombination of electrons and holes. The nature and concentration of the dopant change the charge distribution on the TiO_2_ surface and affects the process of photo corrosion and photocatalytic activity [20]. However, an increase in the absorption of visible light does not always lead to an increase in the activity of the photocatalyst. As a result of doping with cations, a certain number of defects appear in the TiO_2_ structure, which can act as charge recombination centers, this leading to a decrease in photocatalytic activity even under the influence of UV light.

In [21], Kryzhitsky et al. show the change in the activity of photocatalytic properties of rutile and anatase forms of nanocrystalline TiO_2_ depending on the nature of metal-based dopants. According to the results of the study, it is found that doping does not significantly change the bandgap of rutile, while in the case of doping anatase with iron and chromium, its bandgap narrows significantly. As a result of doping with metals, the photocatalytic activity of anatase (A) increases in the following order: A < A/Co < A/Cu < A/Fe. In the case of rutile (R), its photocatalytic activity decreases in the following order: R > R/Co > R/Cu > R/Fe > R/Cr. According to the authors, the decrease in the photoactivity of TiO_2_ may be associated with the inhibitory effect of impurity cations.

### 2.4. Doping with Anionic Elements

Over the past few years, it has been shown that TiO_2_ samples doped with nonmetallic elements (nitrogen, carbon, sulfur, boron, phosphorus, and fluorine) in the anionic positions of TiO_2_, demonstrate high photoactivity in the UV and visible regions of the solar spectrum [22,23]. Among all the anions, carbon, nitrogen, and fluorine caused the most significant interest [24,25,26]. The substitution of oxygen atoms to carbon leads to the formation of new levels (C2p) above the ceiling of the valence band of TiO_2_ (O2p), which reduces the bandgap and shifts the absorption spectrum. The inclusion of carbon in TiO_2_ can also lead to the formation of carbon compounds on the surface of the photocatalyst, which acts as absorption centers of visible radiation [27].

**Figure 3:**
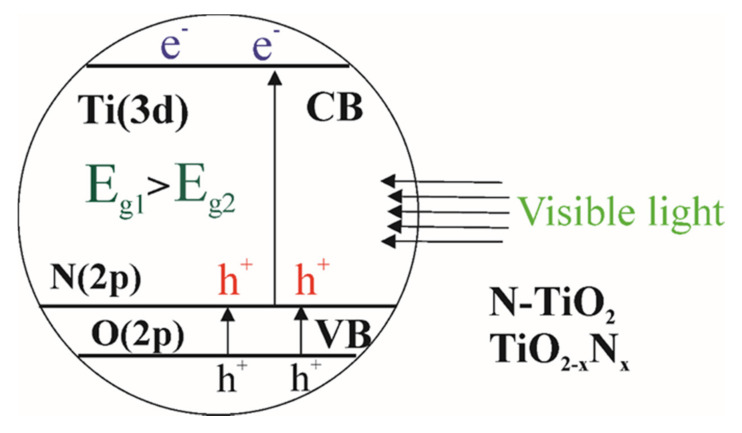
Changes in the band gap of TiO_2_ upon doping with nitrogen atoms.

Doping with nitrogen atoms is the most popular way to improve the photocatalytic performance of TiO_2_. Introduction of nitrogen into the TiO_2_ structure contributes to a significant shift of the absorption spectrum into the visible region of the solar spectrum, a change in the refractive index, an increase in hardness, electrical conductivity, elastic modulus, and photocatalytic activity in regard to visible light [28,29]. Upon substitution of anions, a new level is formed above the valence band of TiO_2_ [30]. As shown in Figure 3, the presence of nitrogen leads to a change of the bandgap E_g1_ (TiO_2_) > E_g2_ (N-doped TiO_2_), thus contributing to the absorption of photons of light with lower energy.

Doping with nitrogen in the oxygen position is difficult since the ionic radius of nitrogen (1.71 Å) is much larger than that for oxygen (1.4 Å). Another auspicious element in the anionic positions of TiO_2_ is fluorine atoms [31]. Unlike nitrogen, fluorine atoms easily replace oxygen due to the close ion radius (1.33 Å for F^−^ and 1.4 Å for O^2−^). The increase in photocatalytic activity is mainly associated with an improvement in the degree of crystallinity of TiO_2_ due to doping with fluorine [32]. It has been determined that crystallinity and the specific surface area also affect the photoactivity of TiO_2_ [33,34]. The crystalline modification of TiO_2_ in comparison with amorphous TiO_2_ has significantly fewer defects, which reduce the possibility of recombination processes and contributes to the efficient movement of photogenerated charge carriers in the semiconductor. Since redox reactions occur on the surface of TiO_2_, one of the main requirements for photocatalysts is the presence of a developed specific surface area. However, the presence of a developed specific surface area implies a large number of defects in the structure and a low degree of crystallinity and, as a result, reduces photocatalytic activity. Therefore, to increase photocatalytic activity, it is important to find a balance between the above factors.

### 2.5. Doping/Loading with Metal Nanoparticles

The application of metal nanoparticles is another alternative approach to the modification of photocatalysts. A review of the recent literature shows that metals (Co, Pt, Ag, Au, Pd, Ni, Cu, Eu, Fe, etc.) significantly increase the photocatalytic activity of TiO_2_ [35,36,37]. The low location of the Fermi level of these metals compared to TiO_2_ can lead to the movement of electrons from the TiO_2_ structure to metal particles deposited on its surface. This helps to avoid the recombination of charge carriers, since the holes remain in the valence band of TiO_2_. This is also beneficial for avoiding the recombination of charge carriers since the holes remain in the valence band of TiO_2_. A number of conducted investigations indicate that the properties of these photocatalysts depend on the dispersion of metal particles [38,39]. Enhanced photocatalytic properties of metals appear when their size decreases <2.0 nm [40]. Despite the foregoing, too high concentration of metal particles can block the surface of TiO_2_ and prevent the absorption of photons, leading to a decrease in the efficiency of the photocatalyst.

## 3. The Utilization of Photocatalysts Based on TiO_2_

### 3.1. Hydrogen Evolution

Hydrogen can be produced by using nanoscale TiO_2_ based photocatalysts with various morphologies in the form of nanowires, nanospheres, nanorods, nanotubes, and nanosheets [41]. Table 1 lists some TiO_2_ nanocomposites with different structures, as well as their photocatalytic characteristics.

P. Melián et al. [42] demonstrated that loading of nanosized TiO_2_ microspheres with Au and Pt metals increases the hydrogen evolution twice. In the case of using Au, the maximum hydrogen evolution was determined at 1.5 wt.% content of Au in the sample with the yield of hydrogen 1118 µmol h^−1^, while for Pt its optimal content in the sample was 0.27 wt.% with the yield of hydrogen 2125 µmol h^−1^. The excess of dopants may result in decrease of photocatalyst activity due to possible complete coverage of TiO_2_ surface, thus, hindering the light to be absorbed. A literature review also showed that the difference in the optimal ratio for each metal could be associated with the formation of recombination centers on the semiconductor surface by metal particles [51,52].

**Figure 4:**
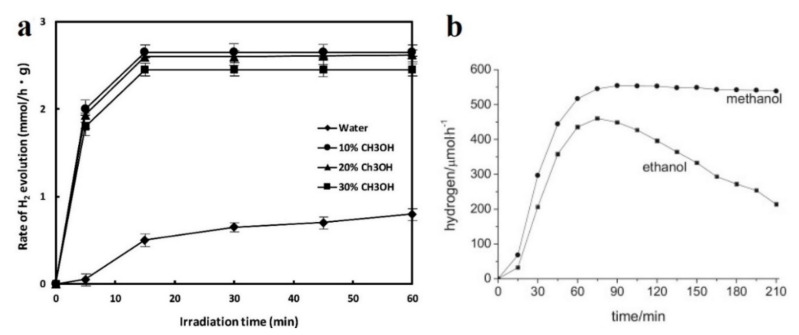
(**a**) Rate of hydrogen evolution from photocatalysis of aqueous methanol solution on Ag/TiO_2_ photocatalysts. This figure is reprinted from [60], with permission from Elsevier, 2020; (**b**) H_2_ production patterns for 24.47 M (100% *v/v*) of methanol and 17.06 M (100% *v/v*) of ethanol. This figure is reprinted from [54], with permission from Elsevier, 2015.

A positive effect on the rate of H_2_ production was found when using sacrificial agents acting as electron donors (hole scavengers) during photoreforming, in which the hydroxyl radical is consumed by the sacrificial agents. In general, there are two types of sacrificial reagents: organic and inorganic based electron donors. Among organic electron donors, the most effective are water–alcohol mixtures, in particular, methanol > ethanol > ethylene glycol > glycerol [53,54,55,56,57,58,59]. However, an increase in the concentration of the sacrificial agent does not always lead to an increase in the yield of hydrogen. Y.-K. Park et al. [60] showed that the rate of hydrogen evolution also increases depending on the concentration of methanol (Figure 4a). At low concentrations, the rate of hydrogen formation in solutions is proportional to the concentration of methanol, while at higher concentrations, it approaches to a constant value [61]. Nevertheless, after adding a certain amount of methanol and ethanol, a further increase in its concentration leads to a decrease in the rate of hydrogen evolution (Figure 4b). The yield of hydrogen during photoreforming has a maximum output during 80−90 min and after it decreases. This is due to the formation of a significant amount of methane and ethane, during which photogenerated electrons ($e_{CB}^{-}$) and holes ($h_{VB}^{+}$) are consumed [54].

**Figure 5:**
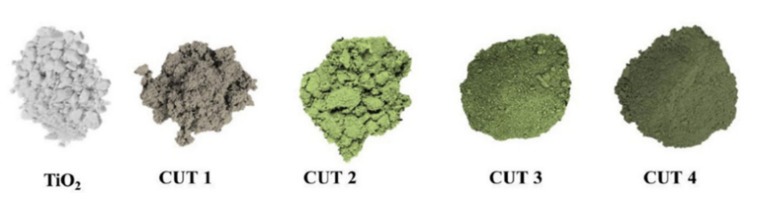
Colour of synthesized pristine and Cu-TiO_2_/CuO composite nanopowders. This figure is reprinted from [62], with permission from Elsevier, 2020.

The amount of dopant also has a significant effect on the efficiency of light absorption by the photocatalyst and on its photocatalytic activity. For example, Udayabhanu et al. [62] prepared Cu-TiO_2_/CuO nanocomposites containing different amount of Cu. The color of the obtained samples, depending on the concentration of Cu-TiO_2_/CuO (CUT) from 1 to 4 mol% (the samples were as named CUT 1, CUT 2, CUT 3, and CUT 4) changes from light green to dark green (Figure 5). To evaluate the photocatalytic activity of hydrogen production, scientists compared the activities of obtained nanocomposites with conventional TiO_2_. Sunlight was used as the source of radiation, and glycerol was chosen as a sacrificial agent. According to the results, the CUT 3 sample based on Cu-TiO_2_/CuO possessed the highest yield of hydrogen (10.453 µmol h^−1^ g^−1^ of H_2_ under sunlight and 4.714 µmol h^−1^ g^−1^ of H_2_ under visible light). Nevertheless, this type of photocatalyst is effective only for hydrogen production, since it has shown low efficiency in the decomposition of organic dye and metal detoxification in water.

**Figure 6:**
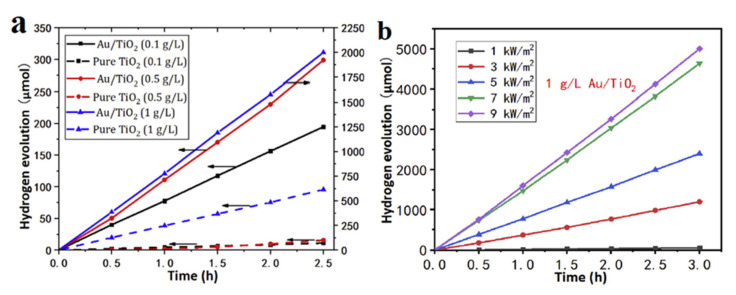
(**a**) Hydrogen evolution of pure TiO_2_ nanoparticles and Au/TiO_2_ nanoparticles at the same light intensity of 5 kW/m^2^; (**b**) Hydrogen evolution in 1–9 kW/m^2^ light intensities of 1 g/L Au/TiO_2_ solutions. Both figures are reprinted from [63], with permission from Elsevier, 2020.

The photocatalyst efficiency is affected not only by the nature of the alloying element but also by its concentration. For example, X. Xing et al. in [63] demonstrated the dependence of the yield of hydrogen on the light intensity and the concentration of the dopant. From Figure 6a, it is clear that the increase of concentrations of each photocatalyst (pure TiO_2_ and Au/TiO_2_) results in increase of the rate of hydrogen production. However, the hydrogen generation rate for Au/TiO_2_ photocatalyst at the same light intensity is 18–21 times higher than that for pure TiO_2_. The explanation to this is that Au can limit charge carriers’ recombinations and, what is more, the visible light absorption of photocatalyst is enhanced by the localized surface plasmon resonance effect of Au nanosized particles. The influence of the light intensity (from 1 to 9 kW/m^2^) and the duration of exposure on the rate of hydrogen generation for Au/TiO_2_ nanoparticles with a concentration of 1 g/L is also shown in Figure 6b.

The photocatalytic properties of the material can be improved by creating composites based on different photocatalysts. For example, E.-C. Su et al. [64] obtained a composite photocatalyst based on Pt/N-TiO_2_/SrTiO_3_-TiO_2_ in the form of nanotubes using a two-stage hydrothermal process (SrTiO_3_ is also a photocatalyst with a bandgap of 3.2 eV with a perovskite-type structure [65]). The obtained results showed that this composite photocatalyst is able to operate under sunlight with the rate of hydrogen evolution up to 3873 µmol/h/g.

In practice, photocatalytic reactions are mainly carried out at room temperature. It is found that increasing temperature has a positive effect on the activity of some photocatalysts, which makes it relevant to develop new photocatalysts based on the anatase form of TiO_2_ with a thermostable phase.

### 3.2. Photodegradation

Organic dyes are used on a large scale in modern industries. Wastewater polluted with such substances subsequently leads to environmental problems [66,67]. This is due to the fact that most organic dyes consist of biodegradable aromatic structures and azo-groups. Adsorption and catalytic oxidation remain the most effective among the methods for treating wastewater from dyes [68,69,70]. For photocatalytic degradation of organic compounds, in most cases, TiO_2_, in the doped form, is used.

Graphene has a large specific surface area and electrical conductivity. The presence of such properties in graphene is of interest to scientists in the preparation of a graphene/nano-TiO_2_-based photocatalyst [71,72,73]. After irradiation of a TiO_2_-based photocatalyst, electrons move into the graphene structure. Based on the author’s results [74], it helps to avoid recombination between charge carriers. TiO_2_/graphene-based photocatalyst can be obtained by creating either a chemical bond between them or via the approach of their mechanically mixing [75,76]. Due to existence of chemical bond, the electron is transferred unhindered from the photocatalyst to graphene, reducing the probability of recombination. This is the main explanation for the increased activity of the photocatalyst with graphene.

Composites based on TiO_2_/graphene attract scientists’ attention not only as highly efficient photocatalysts due to light absorption in a wide range and charge separation, but also taking in account its high adsorption capacity to pollutants. However, TiO_2_/graphene-based composite produced by the hydrothermal method cannot serve as an effective photocatalyst. The reason is the agglomeration of graphene layers, which adversely affects the adsorption and photocatalytic properties. The solution to this problem is described in [77], where graphene-based aerogel was used as an auxiliary material for TiO_2_ [78,79]. Aerogel was obtained by thermal reduction of graphene oxide. Table 2 lists some reports on the use of composite TiO_2_/graphene based photocatalysts to remove various organic compounds (pollutants and dyes) from water.

**Figure 7:**
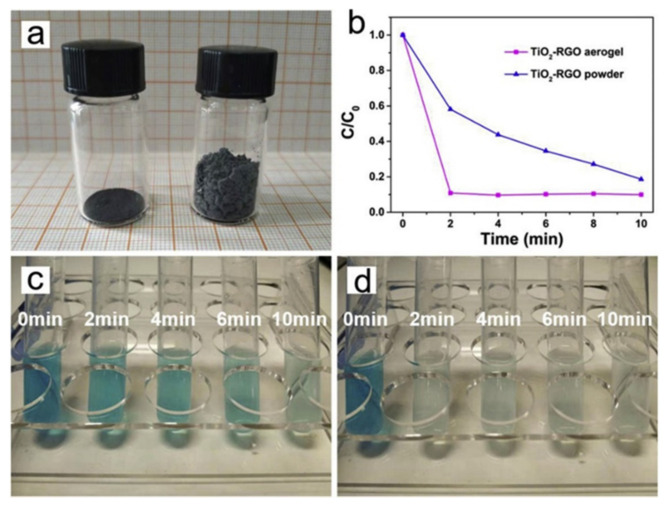
(**a**) Photograph of 80 mg TiO_2_-reduced graphene oxide (rGO) powder (left) and aerogel (right); (**b**) methylene blue (MB) dark adsorption results of the TiO_2_-rGO aerogel/powder; color changing of MB solution during dark adsorption with TiO_2_-rGO powder (**c**), and aerogel (**d**). All figures are reprinted from [77], with permission from Elsevier, 2020.

Before testing photocatalytic activity, it was necessary to saturate the materials in the dark conditions. If saturation is not performed at dark conditions, a decrease in the concentration of the target pollutant or the color intensity change of the used in experiment dye will be associated not only with photocatalysis, but also with adsorption and photocatalytic degradation, so the effectiveness of the photocatalyst will be incorrect. X. Sun et al. [77] compared two samples of a photocatalyst of the same mass: hydrothermally obtained TiO_2_-reduced graphene oxide (rGO), and aerogel based on TiO_2_-rGO (Figure 7a). As can be seen, despite the equal mass of both samples, the TiO_2_-rGO based aerogel has a larger specific volume than that of the second sample. For further characterization of their adsorption, both samples were used to adsorb methylene blue in the dark. During the experiment, the absorption intensity was analyzed. The results showed that it took 2 min for TiO_2_-rGO based aerogel to achieve adsorption saturation, while for TiO_2_-rGO powder, it took more than 10 min (Figure 7b). Figure 7c,d demonstrates the color change of methylene blue, which proves the high absorption coefficient of visible light by TiO_2_-rGO aerogels. According to the authors, the adsorption rate also affects the efficiency of photocatalysts.

**Figure 8:**
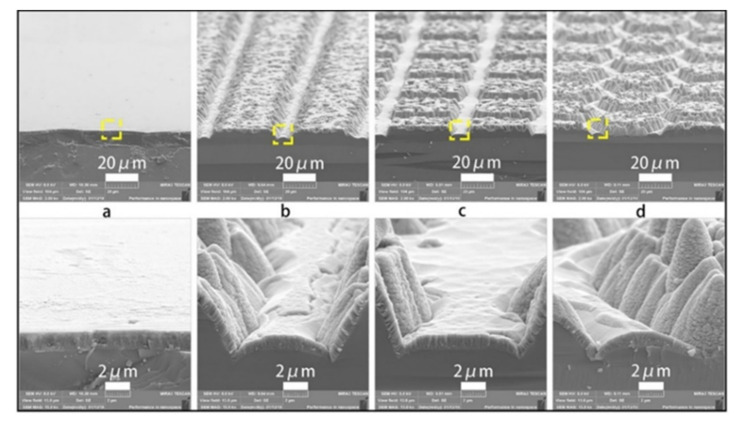
SEM images of (**a**) planar TiO_2_ film; (**b**) grating-structured TiO_2_ film; (**c**) square-structured TiO_2_ film; (**d**) hexagon-structured TiO_2_ film. All figures are reprinted from [89], with permission from Elsevier, 2019.

Photocatalytic activity directly depends on the number of active centers on the surface of the photocatalyst. Photolithography is a block of technological processes of photochemical technology aimed at creating the relief in the film, as well as a film of metal deposited on a substrate [87,88]. Using this method, a group of scientists [89] managed to obtain TiO_2_ films with lattice, square, and hexagonal structures (Figure 8) and investigate the influence of a such surface textures on the photocatalysis. Obtained results showed that the activity of photocatalyst in the form of a film is not improved by increasing the values of specific surface area. Surface texture also has an effect on mass transfer during photocatalysis.

**Figure 9:**
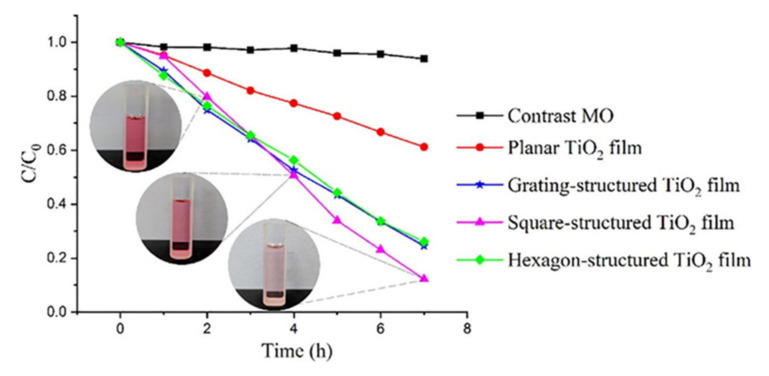
Photocatalytic degradation of MO under ultraviolet light (254 nm) irradiation. C and C_0_ represent the real-time and initial concentration of methyl orange solution. This figure is reprinted from [89], with permission from Elsevier, 2019.

To evaluate the activity of photocatalysts, scientists conducted an experiment, in which the dye degradation occurred in water as a result of its exposure with UV (254 nm) on methyl orange. As shown in Figure 9, the microstructured TiO_2_ films exhibit a more active photocatalytic activity than TiO_2_ films with a flat surface. According to scientists, this is due to the presence of reaction centers on the surface of microstructured films. The highest photocatalytic activity was observed for TiO_2_ films with a square microstructure. The experimental results showed that the efficiency of photocatalysts is influenced not only by the surface area, but also by the type of their microstructure. It was revealed, that the TiO_2_ film with a grating structure, despite its low specific surface area, possessed photoactive properties similar to the TiO_2_ film with a hexagonal structure [89].

A significant role during photocatalysis is played by the surface microstructure, which influences the mass transfer of degradable organic compounds by the diffusion to the surface of the photocatalyst. It is important to take into account the fact that the fluid flowing over the surface with protrusions meets more resistance from the side of the walls, compared to the fluid flowing through the flat surface. As a result, such protrusions can adversely affect the degradation efficiency of organic pollutants.

**Figure 10:**
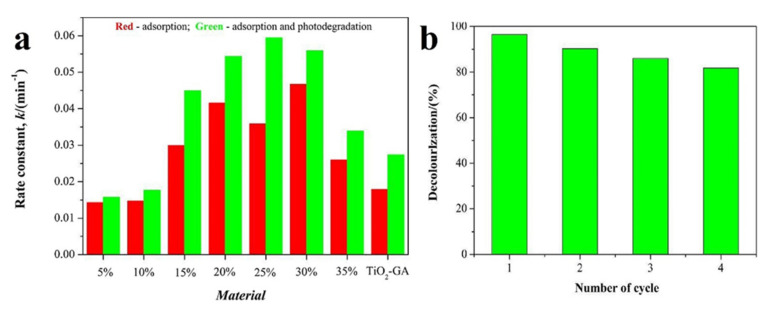
(**a**) The rate constants for the adsorption and the collective removal of rhodamine B (RhB) over TiO_2_-GA and the Fe_2_O_3_-TiO_2_-GA composites; (**b**) stability of the Fe_2_O_3_-TiO_2_-GA (25%) composites in the removal of RhB dye. Both figures are reprinted from [91], with permission from Elsevier, 2018.

TiO_2_ doped with Fe_2_O_3_ is one of the best-known effective photocatalysts. Due to the narrow bandgap of Fe_2_O_3_ (2.2 eV), its doping leads to a redshift of the light response of the photocatalyst. The phenomenon of decreasing of photoactivity of TiO_2_ based photocatalyst doped with non-metals during heating, the high cost of some metals and the availability of Fe_2_O_3_ in large quantities increases the attractiveness of Fe_2_O_3_ over other dopants [90]. J.-J. Zhang et al. [91] used Fe_2_O_3_ nanoparticles, which served as a doping agent for obtaining the TiO_2_/graphene aerogel (GA) based photocatalyst with a 3D structure (Figure 10). Due to the narrow bandgap (2.0 eV), Fe_2_O_3_ can easily generate electron-hole pairs, thereby contributing to the photodegradation of rhodamine B even in visible light. The results showed that the aqueous solution containing rhodamine B (RhB) was purified to 97.7% (Figure 10 b). Fe_3_O_4_ can also be used as a doping agent for the photocatalysis [92]. For example, F. Soltani-Nezhad et al. [93] presented a method for producing a GO/Fe_3_O_4_/TiO_2_-NiO-based photocatalyst, which is able to efficiently degrade imidacloprid (pesticide).

To increase the efficiency of the process of degradation of organic pollutants by photocatalysis, attempts have been made to combine radiation with ultrasonic cavitation. S. Rajoriya et al. [94] reported the photodegradation of 4-acetamidophenol to 91% using Sm (samarium) and N-doped TiO_2_ photocatalysts, in which a combination of UV radiation, hydrodynamic cavitation, and ultrasound was applied. In another work, the use of N and Cu-doped TiO_2_@CNTs in sono-photocatalysis for purification of pharmaceutical wastewater is discussed [95]. The results showed that when using a commercial Xe lamp (50 W) and ultrasound for pharmaceutical wastewaters treatment, the photocatalyst removal efficiency within 180 min were 100, 93, and 89% for sulfamethoxazole, chemical oxygen demand, and total organic carbon, respectively.

The great interest in the use of ultrasonic action during photocatalysis is justified by the enhancement of electronic excitations, which leads to an increase in the density of pairs of charged particles. Under the influence of ultrasound, the aggregate photocatalysts are dispersed, contributing to the rapid renewal and expansion of the boundaries of heterogeneous reactions, which improves the mass transfer and the course of chemical reactions [96,97].

## 4. Conclusions

In this review, information on the main mechanisms of water splitting and decomposition of organic compounds by TiO_2_-based photocatalysts was collected and analyzed. The efficiency of TiO_2_ doping by various components, which significantly increases the photocatalytic activity, is shown. In addition, other factors, such as the bandgap, charge carrier recombination, the use of sacrificial agents, the size and type of photocatalyst structures, surface morphology, photocatalyst concentration in solution, radiation source power, etc., which can affect the photocatalysis were analyzed. Despite the reports of scientists on the development of effective photocatalysts capable of operating under sunlight, their use is still not economically viable. Therefore, due to the growth of environmental problems and the limited availability of hydrocarbon fuels, investigations in the field of hydrogen production by “artificial photosynthesis” will undoubtedly increase.

## Figures and Tables

**Table 1:** TiO_2_-based photocatalysts with different structures utilized for hydrogen evolution under splitting water mixtures.

Photocatalyst	Structure	Light Source	Sacrificial Agents	Evolution H_2_	Ref.
Au/TiO_2_ Pt/TiO_2_	microspheres	15 W fluorescent tubes (λ_max_ = 365 nm)	25% vol. methanol	1118 µmol h^−1^ 2125 µmol h^−1^	[42]
TiO_2_	nanofibers	300 W Xenon lamp	10% vol. methanol	3200 μmol h^−1^ g^−1^	[43]
Cu/TiO_2_	nanorods	300 W Xe lamp (λ > 300 nm)	20% vol. methanol	1023.8 μmol h^−1^	[44]
TiO_2_/WO_3_/Au	nanofibers	300 W Xe arc lamp	35% vol. methanol	269.63 µmol h^−1^	[45]
M/TiO_2_/rGO M = Au or Pt	nanoparticles	300 W Xenon lamp (λ > 300 nm)	20% vol. methanol	670 µmol h^−1^	[46]
MoSe_2_/TiO_2_	nanoparticles	Xe arc lamp (PLS-SXE300)	10% vol. methanol	4.9 μmol h^−1^	[47]
BCN-TiO_2_	nanosheets& nanoparticles	300 W xenon lamp with a UV-cutoff filter (λ ≥ 420 nm)	20% vol. triethanolamine	68.54 μmol h^−1^ g^−1^	[48]
TiO_2_/C_3_N_4_	double-shell microtubes	300 W xenon lamp	20% vol. methanol	10.1 mmol h^−1^ g^−1^	[49]
ZnS@g-C_3_N_4_/TiO_2_	nanospheres	300 W Xenon lamp (λ > 400 nm)	10% vol. triethanolamine	422 μmol h^−1^ g^−1^	[50]

**Table 2:** Removal percentage of some organic pollutants by TiO_2_/graphene based photocatalysts.

Photocatalyst	Organic Pollutant	Light Source	Irradiation Time	Efficiency	Ref.
TiO_2_@rGO	2,4,6 trichlorophenol	Mercury lamp (11 W)	180 min	90%	[80]
TiO_2_/Fe_3_O_4_/GO	Methylene blue	Halogen lamp (500 W)	90 min	76%	[81]
GO/TiO_2_ nanotubes	Perfluorooctanoic acid	UV lamp (8 W)	240 min	97%	[82]
N-TiO_2_/Ag_3_PO_4_@GO	Acid Blue 25	Halogen bulb (250 W)	20 min	98%	[83]
Ag and rGO modified TiO_2_	Tetrabromobisphenol A	Xenon light (500 W)	80 min	99.6%	[84]
N-doped graphene/TiO_2_	Bisphenol A	Mercury lamp (300 W)	60 min	100%	[85]
3D polyaniline/TiO_2_/rGO hydrogel	BPA	Mercury lamp (500 W)	40 min	100%	[86]
